# Albumin Modifies Responses to Hematopoietic Stem Cell Mobilizing Agents in Mice

**DOI:** 10.3390/cells9010004

**Published:** 2019-12-18

**Authors:** Eva Danner, Halvard Bonig, Eliza Wiercinska

**Affiliations:** 1 German Red Cross Blood Donor Service Baden-Wuerttemberg-Hesse, 60528 Frankfurt, Germany; e.danner@blutspende.de (E.D.); h.boenig@blutspende.de (H.B.); 2 Faculty of Biological Sciences, Goethe University, 60438 Frankfurt, Germany; 3 Goethe University Medical School, Institute for Transfusion Medicine and Immunohematology, 60528 Frankfurt, Germany

**Keywords:** hematopoietic stem cell mobilization, hematopoietic stem cell transplantation, granulocyte-colony stimulating factor, serum albumin deficiency, pharmacodynamics, AMD3100, Plerixafor

## Abstract

Albumin, the most abundant plasma protein, not only controls osmotic blood pressure, but also serves as a carrier for various small molecules, including pharmaceuticals. Its impact on pharmacological properties of many drugs has been extensively studied over decades. Here, we focus on its interaction with the following mobilizing agents: Granulocyte-colony stimulating factor (G-CSF) and AMD3100, where such analyses are lacking. These compounds are widely used for hematopoietic stem cell mobilization of healthy donors or patients. Using albumin-deficient (Alb−/−) mice, we studied the contribution of albumin to mobilization outcomes. Mobilization with the bicyclam CXCR4 antagonist AMD3100 was attenuated in Alb−/− mice compared to wild-type littermates. By contrast, mobilization with recombinant human G-CSF (rhG-CSF), administered twice daily over a five-day course, was significantly increased in Alb−/− mice. In terms of a mechanism, we show that rhG-CSF bioavailability in the bone marrow is significantly improved in Alb−/− mice, compared to wild-type (WT) littermates, where rhG-CSF levels dramatically drop within a few hours of the injection. These observations likely explain the favorable mobilization outcomes with split-dose versus single-dose administration of rhG-CSF to healthy donors.

## 1. Introduction

Albumin is, by a wide margin, the most abundant plasma protein of all higher species, including man, typically at concentrations of 30–50 g/L. It not only controls colloid osmotic pressure [1,2,3] but also serves as a depot and a carrier for both endogenous small molecules and medicinal substances [4]. Its structure was described in the 1970s [5] and binding pockets were characterized shortly after [6]. Due to its high binding potential for a broad range of substances, serum albumin is an obvious frequent focus of pharmacokinetic studies [6,7,8]. Until recently, the lack of animal models for studying pharmaceutical properties of albumin-binding drugs restricted experiments to in vitro models, which, however, were inadequately predictive. In 2015, the group of Michael Wiles developed a mouse model that lacked endogenous albumin [9]. The model was intended to facilitate substitution of mice with human serum albumin, which increases albumin half-life, from 2.6 to 4.2 days, thus allowing for the study of drug binding to human albumin in mice. Albumin deficient (Alb−/−) mice are viable and healthy; they compensate for the lack of serum albumin by increasing plasma levels of triglycerides, cholesterol, and aspartate aminotransferase. Nevertheless, the total serum protein level is still significantly lower than in wild-type mice and comparable to hypoalbuminemic patients [9,10].

Hematopoiesis is the continuous, life-long process of generating short-lived mature blood cells that originate from hematopoietic stem and progenitor cells (HSPC). The process of proliferation and cell differentiation is tightly controlled. Individual HSPC will undergo asymmetric cell divisions, whereby progenitors arise. Under the influence of their environment in the bone marrow (BM), they will give rise to increasingly more differentiated precursors and mature blood cells, which are ultimately released into the circulation [11]. The near-quantitative retention of HSPC in the BM is significantly, albeit not exclusively, controlled by the interaction of stroma-derived C-X-C motif chemokine ligand 12 (CXCL12), with C-X-C chemokine receptor type 4 (CXCR4), which is expressed on the surface of HSPC [12,13].

Our interest in HSPC is significantly driven by translational aspects. In particular, the enforced egress of HSPC, so called mobilization, for graft manufacturing, remains a focus of our studies. HSPC are routinely collected from the peripheral blood of healthy donors after a 5-day course of mobilization with recombinant human granulocyte-colony stimulating factor (rhG-CSF) [14,15]. As a small glycoprotein, rhG-CSF could potentially bind to albumin, and thus be either stabilized and transported through the blood stream, or functionally neutralized. The exact mechanism of action of G-CSF in the BM during mobilization is still not completely understood, but evidence was presented that, at first, HSPC are forced by G-CSF to proliferate [16,17,18,19,20]. Later on, a proteolytic milieu, possibly established by G-CSF-activated neutrophils, cleaves retention factors, allowing for HSPC egress [11,21]. Moreover, a plethora of other cellular G-CSF targets within the BM niche was suggested (osteoblasts [22], osteocytes [23], CXCL12-abundant reticular cells [24], nestin-positive mesenchymal stromal cells [25], or osteo-macs [26] to name a few). Thus, it is conceivable that the need for orchestrated activation of multiple niche components contributes to the rather slow kinetics of G-CSF mobilization. In contrast, AMD3100 can be used as a bolus injection to mobilize HSPC. It is a direct CXCR4 antagonist, disrupting the CXCR4–CXCL12 axis and activating proteases; thus, leading to a more rapid (within hours), albeit much less efficient mobilization than G-CSF [27]. AMD3100 is approved for use in combination with G-CSF in G-CSF refractory patients. Pharmacokinetic studies showed a half-life of 4–5 h for AMD3100 [28] and 4–22 h for G-CSF in human blood [29,30], but how these relate to the biological half-life is entirely unclear, as pharmacodynamics effects are, by comparison, much protracted. Development of rhG-CSF and AMD3100 preceding the Wiles’ mouse, studies in the absence of albumin, were not previously performed.

Serum albumin plays a crucial role in the transport of small molecules and drugs through the blood stream, and as such is a potential carrier of both AMD3100 and rhG-CSF. As was shown, up to 58% of administered AMD3100 directly binds to plasma proteins [31] including albumin. A potential impact of albumin on rhG-CSF bioavailability was not previously studied. Albumin null mice gave us the opportunity to test for responsiveness of HSPC to mobilizing agents in the absence of albumin, and to test specificity of the observations by substituting albumin prior to mobilization. In the course of this study, we demonstrate a negative impact of human serum albumin on the tissue distribution of rhG-CSF. Inversely, analbuminemia strongly enhances the mobilization efficiency.

## 2. Materials and Methods

### 2.1. Mice

For all experiments, young adult mice (8–12 weeks, male or female) were used. C57BL/6J-Alb^em8Mvw^/MvwJ (Alb−/−) mice were described previously [9], albeit not with respect to their hematopoietic phenotype, and were purchased from Jackson Laboratory (JAX stock #025200, Sulzfeld, Germany). Albumin-competent wild-type (WT) littermates of the corresponding strain were used as controls. All experiments were performed in agreement with the German Animal Welfare Act and approved by the municipal government (F27/1004, Regierungspraesidium Darmstadt, Darmstadt, Germany).

### 2.2. Hematopoietic Cells

Peripheral blood was drawn from the facial vein into EDTA tubes using a 23G needle. Total cell counts were analyzed by Hemavet 950SF+ (Drew Scientific, Dallas, TX, USA). BM cells were harvested by aseptically flushing femurs and/or tibiae using Phosphate-Buffered Saline (PBS) + 0.5% Bovine Serum Albumin (BSA). Splenic cells were obtained by aseptic blunt extrusion of the capsule.

### 2.3. Enumeration of Hematopoietic Cells

Hematopoietic cells in blood, BM, and spleen were enumerated using multi-parametric flow cytometry for informative markers, for mature and immature subsets, as well as in vitro clonogenic assays [32,33,34]. Antibodies used are listed in Supplemental Table S1; acquisition was done with FACS LSR Fortessa (Becton-Dickinson, Heidelberg, Germany) and analysis with FACSDiva 7 (Becton-Dickinson, Heidelberg, Germany). Clonogenic assays were performed with cytokine-replete semisolid culture media (Methocult GF M3434, Stem Cell Technologies, Cologne, Germany). A defined aliquot of white blood cells (WBCs) from the different tissues (after hypotonic red blood cell lysis for peripheral blood) was plated in duplicate, and colony growth was scored after 7 days using an inverted 2.5× microscope (Olympus, Hamburg, Germany). Cell cycle analysis was performed using Ki67 as a marker for cell proliferation. 7-AAD was added to distinguish between G1 and G2/S/M phases.

### 2.4. Mobilization

Progenitor cells were mobilized into peripheral blood using either AMD3100 (5 mg/kg bolus, i.p., Sigma-Aldrich, Darmstadt, Germany) or rhG-CSF (nine doses of 100 µg/kg q12h i.p., Sandoz-Hexal, Holzkirchen, Germany) either in 0.9% NaCl (BBraun, Melsungen, Germany) or 20% human albumin solution (Baxter, Deerfield, IL, USA). Blood was drawn 1 h, 2 h, and 4 h after administration of AMD3100 or 1 h after the last administration of rhG-CSF (unless otherwise stated), followed by colony-forming unit culture (CFU-C) enumeration in blood, as well as, where indicated, in BM and spleen. WBC were counted at every time point. LSK, LSK-SLAM, and cell cycle analysis were performed in BM and spleen cell suspensions using flow cytometry.

### 2.5. Albumin Quantification

Human serum albumin in mouse serum was detected and quantified using human albumin ELISA (Merck, Darmstadt, Germany) according to the manufacturer’s recommendations.

### 2.6. rhG-CSF Quantification

Human G-CSF Flex Set (BD Biosciences, Franklin Lakes, NJ, USA) was used to quantify levels of rhG-CSF in murine plasma or BM-fluid samples.

### 2.7. Statistics

Descriptive statistics and students’ *t*-tests were calculated using Excel (Microsoft, Redmond, WA, USA); twoway ANOVA with Bonferroni post-test was calculated using GraphPad Prism 5 (GraphPad Software, Inc., La Jolla, CA, USA); for non-normally distributed data, the nonparametric Mann–Whitney U test was calculated using SPSS (IBM, Armonk, NY, USA). Unless stated otherwise, results are presented as mean ± standard error of the mean (SEM), ns *p* ≥ 0.05, * *p* < 0.05, ** *p* < 0.01, *** *p* < 0.001.

## 3. Results

### 3.1. Homeostatic Hematopoiesis is Unaffected by Albumin Deficiency

**Figure 1:**
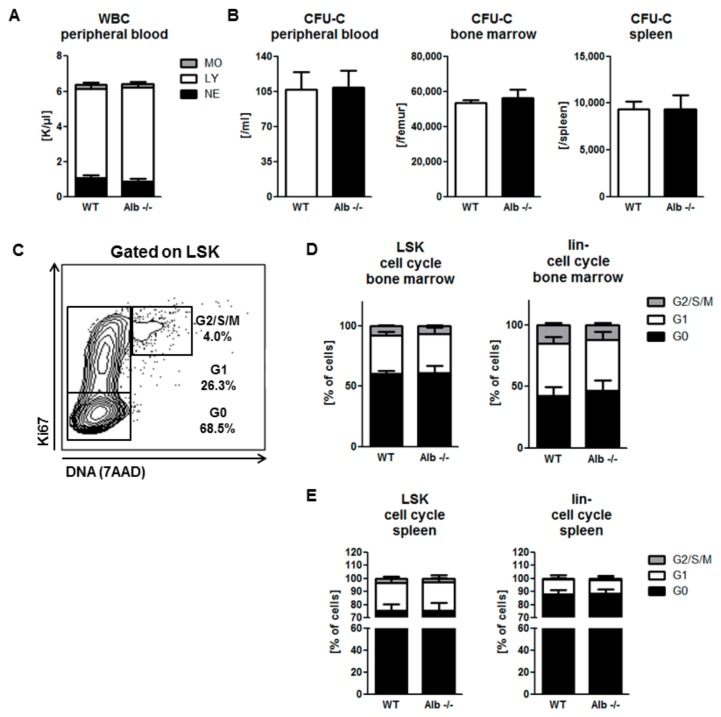
Adult hematopoiesis is unaffected by albumin deficiency. Peripheral blood leukocyte counts and three-way differential were no different in young adult albumin deficient (Alb−/−) mice vs. wild-type (WT) littermates (**A**) MO, monocytes; LY, lymphocytes; NE, neutrophils. Indistinguishable colony-forming unit culture (CFU-C) counts in peripheral blood, bone marrow (BM), and spleen (**B**) FACS-based cell cycle analysis (**C**) Highly similar cell cycle distribution of LSK and lin-cells in BM (**D**) and spleen between the genotypes (**E**) Data from three individual experiments, *n* ≥ 10 mice per group.

In order to assess the effect of albumin deficiency on hematopoietic stem cell mobilization, we first enumerated phenotypically and functionally mature and immature hematopoietic cells in all hematopoietic organs of untreated young adult Alb−/− mice, or wild-type littermates as baseline values. The numbers of mature leukocytes in all compartments (peripheral blood, including differentials, BM, and spleen) were normal in Alb−/− mice (Figure 1A and Figure S1A). Moreover, the number of functional HSPC (CFU-C) in all hematopoietic compartments was unaffected by albumin deficiency (Figure 1B and Figure S1B), whereas the number of phenotypic HSPC (lineage-Sca1+ c-kit+; LSK) was modestly increased in spleens of Alb−/− mice (Figure S1C). Complementing the assessment of overwhelming similarity of homeostatic hematopoiesis of Alb−/− mice, cell cycle analysis of HSPC (example Figure 1C) did not reveal any differences in HSPC-cycling, neither in BM (Figure 1D), nor in spleen (Figure 1E). In summary, we conclude that homeostatic hematopoiesis is normal in Alb−/− mice.

### 3.2. Role of Albumin in the Pharmacodynamics of the Small-Molecule CXCR4 Antagonist AMD3100

**Figure 2:**
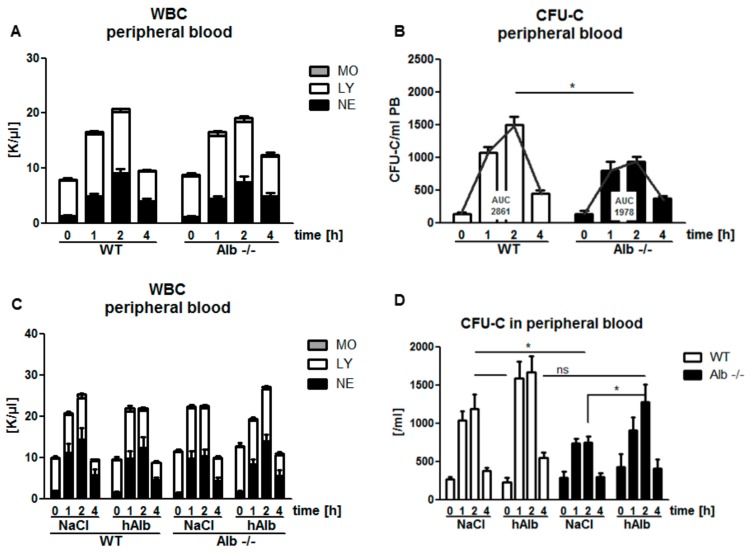
Attenuated hematopoietic stem and progenitor cells (HSPC) mobilization by AMD3100 in Alb−/− mice: Normal mature cell mobilization in Alb−/− mice after AMD3100 treatment (**A**) MO, monocytes; LY, lymphocytes; NE, neutrophils; whereas attenuated HSPC mobilization (CFU-C assay), albeit without affecting pharmacodynamics (**B**) i.v. supplementation with human albumin (hAlb) did not affect white blood cell (WBC) counts in neither genotype (**C**) and largely normalized responsiveness of immature cells (**D**). Data from two to five individual experiments with *n* ≥ 5 mice per group. ns *p* ≥ 0.05, * *p* < 0.05.

Mice received a single i.p. bolus injection of AMD3100. WBC egress and accumulation in peripheral blood could be detected as early as 1 h after administration, with a maximum at 2 h in both Alb−/− and WT mice (Figure 2A) irrespective of genotype. Peripheral blood WBC returned to baseline values within 4 h of AMD3100 administration. In contrast to these observations for mature leukocyte species, Alb−/− mice were abnormal with respect to HSPC mobilization. They were characterized by one-third diminished peak values in Alb−/− mice, despite similar pharmacodynamics in both settings (Figure 2B). Accordingly, the area under the curve (AUC) value for total mobilization efficiency was 30% lower in Alb−/− mice. Remarkably, i.p. substitution of human albumin (hAlb), co-injected together with the AMD3100 bolus did not rescue the effect (data not shown). By contrast, when intravenous hAlb substitution at the same dose preceded the i.p. AMD3100 bolus by as little as 30 min, HSPC mobilization in Alb−/− mice normalized to WT level (Figure 2C,D). Taken together, the presence of human serum albumin facilitates AMD3100 mobilization efficiency, likely by virtue of altering bioavailability and/or pharmacological half-life.

### 3.3. Role of Albumin in G-CSF-Induced Mobilization

**Figure 3:**
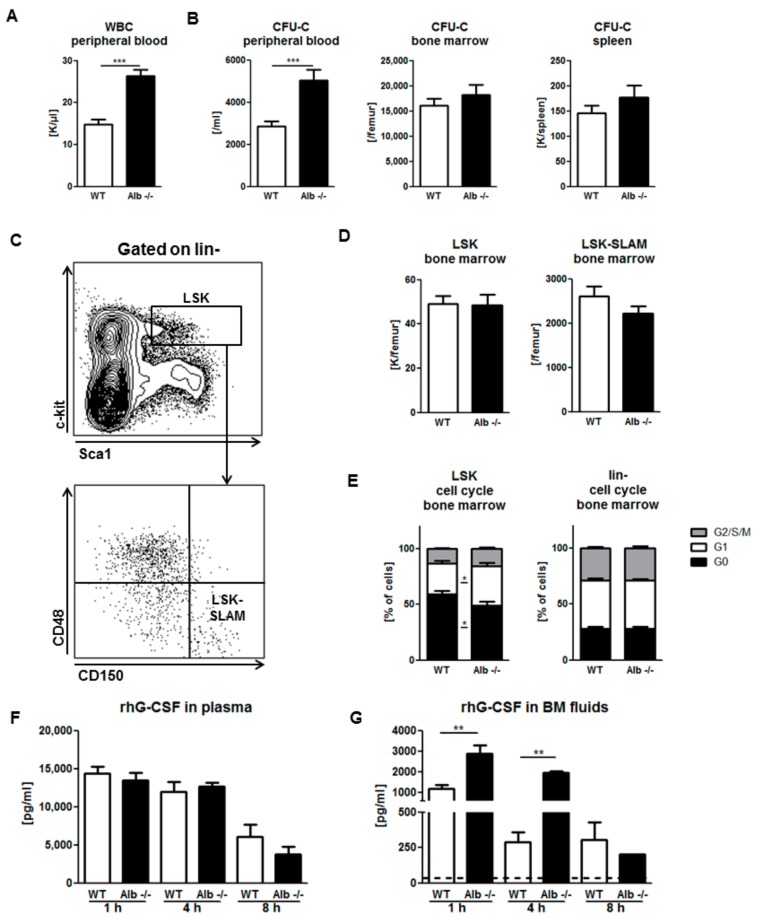
Albumin deficiency enhances Granulocyte-colony stimulating factor (G-CSF)-induced HSPC mobilization: both mature (**A**) and immature (**B**) cell numbers were markedly increased after rhG-CSF treatment in Alb−/− mice compared to WT littermates. Gating strategy for LSK and LSK-SLAM cells (**C**) Number of immature cells in BM and spleen (**B**,**D**) of Alb−/− mice was similar to WT littermates. Significantly, more Alb−/− HSPC were proliferating compared to WT cells (**E**) whereas the effect was gone in the more mature compartment. Data from three individual experiments with *n* ≥ 12 mice per group. Steep gradient of rhG-CSF concentrations between plasma (**F**) and BM fluids (**G**) as well as markedly enhanced and prolonged rhG-CSF accumulation in BM fluids of Alb−/− mice (**G**) Dotted line marks the background (murine plasma without rhG-CSF substitution). Data from two independent experiments with *n* ≥ 3 mice per group and time point. * *p* < 0.05, ** *p* < 0.01, *** *p* < 0.001.

Similarly, modeling clinical mobilization with the more slowly acting cytokine G-CSF, mice received a total of nine, 12-hourly i.p. injections of rhG-CSF, followed by mature and immature leukocyte enumeration in blood, BM, and spleen. Interestingly, albumin-deficient mice showed a 2-fold higher WBC and HSPC mobilization into the circulation, when compared to WT controls (Figure 3A,B and Figure S2A), but the mature cell numbers in the spleen and BM were not affected (Figure S2B). Moreover neither BM progenitor cells (LSK) nor hematopoietic stem cells (HSC: LSK CD48-CD150+, LSK-SLAM) numbers were affected by albumin deficiency (Figure 3C,D). However, HSPC proliferation in the BM of Alb−/− mice upon rhG-CSF treatment was more strongly induced compared to WT littermates (Figure 3E), possibly contributing to the enhanced mobilization. Moreover, splenic HSPC accumulation and HSPC cell cycle activity, after rhG-CSF-treatment of Alb−/− or WT controls, were also indistinguishable (Figure S2C,D).

We hypothesized that local rhG-CSF concentrations might be affected by albumin, and by that mechanism, lead to differences in mobilization efficiency. To test that, we studied the pharmacokinetics of rhG-CSF in plasma and BM fluids of Alb−/− and WT mice. In plasma, we observed the same high (15 ng/mL) and only slowly (over 8 h) decreasing levels of rhG-CSF in both Alb−/− and WT mice (Figure 3F). On the other hand, rhG-CSF peak concentrations in BM were much lower than in blood, and levels were much less sustained: In WT mice, peak levels were 1 ng/mL and 4 h after administration dropped by 70%. In contrast, rhG-CSF BM concentrations in Alb−/− mice reached about 3 ng/mL and were sustained, so that they were 8-fold higher than in WT mice at the four-hour time point (Figure 3G). Thus, albumin impedes accumulation of rhG-CSF in BM, hence resulting in diminished mobilization efficiency.

### 3.4. Human Albumin Substitution in the G-CSF Mobilization Setting

**Figure 4:**
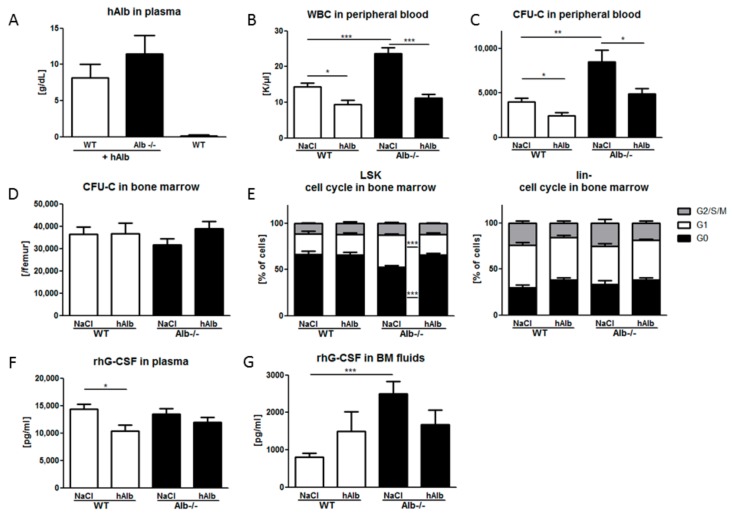
Substitution with hAlb corrects responsiveness to rhG-CSF in Alb−/− mice. Human albumin concentrations were measured one hour after last injection (**A**) showing slightly supra-physiological levels without differences between the genotypes. The hAlb substitution of Alb−/− mice reduced G-CSF-induced mobilization of WBC (**B**) and HSPC (**C**) to WT level. Total CFU-C content in BM was not significantly different between the four groups (**D**) LSK cell cycle activity, slightly increased in the rhG-CSF + NaCl treated Alb−/− mice, was normalized in rhG-CSF + hAlb treated mice (**E**) No such changes were observed in the lin- cell proliferation. The rhG-CSF plasma levels were reduced in WT mice treated with rhG-CSF + hAlb compared to rhG-CSF + NaCl controls (**F**) while rhG-CSF levels in BM fluids after hAlb substitution were comparable between the genotypes (**G**) Data from three individual experiments with *n* ≥ 6 mice per group. * *p* < 0.05, ** *p* < 0.01, *** *p* < 0.001.

To ascertain that the observed mobilization phenotype of the Alb−/− mouse is directly attributable to lack of albumin, as opposed to some complex compensatory mechanism, we also tested rhG-CSF in hAlb-substituted mice of both genotypes, Alb−/− and WT. Bioavailability of hAlb was independent of genotype, so that hAlb substitution raised albumin in Alb−/− mice to near-normal levels (Figure 4A). Endogenous murine, plus substituted human albumin in the WT mice, induced some degree of hyperalbuminemia in the latter (Figure 4A). Thus, in hAlb-substituted mice, HSPC mobilization efficiency was analyzed after a five-day course of rhG-CSF, as described before. Mobilization efficiency in Alb−/− mice was corrected to levels reached by normal WT littermates (Figure 4B,C). Consistent with the impact of albumin substitution on the mobilization in Alb−/− mice, albumin over-supplementation in WT mice also slightly reduced the levels of mobilized WBC and HSPC in the circulation (Figure 4B,C). HSPC counts in BM (Figure 4D) and spleen (Figure S3A) remained unchanged. Cell cycle analysis of BM LSK cells showed a decreased fraction of cells in G1 phase in hAlb-substituted Alb−/− mice (Figure 4E), without similar changes in the more mature lin- compartment. Cell cycle states of LSK cells in spleen were not altered; here, the more mature cells (lin-) were relatively more quiescent after albumin substitution of Alb−/− mice (Figure S3B). Human serum albumin injection markedly reduced plasma rhG-CSF levels in WT mice, while barely affecting those in Alb−/− mice (Figure 4F). In the functionally presumably most relevant compartment for mobilization, however, in BM, after human albumin supplementation, the very low concentrations of rhG-CSF in WT mice were increased, whereas the rather high rhG-CSF concentrations in the Alb−/− mice decreased to essentially similar values in both groups (Figure 4G), albeit the decrease did not reach statistical significance. Thus, we conclude that albumin affects the bioavailability of rhG-CSF in the BM with severe consequences for G-CSF mobilization efficiency.

## 4. Discussion

Albumin is the most abundant serum protein, controlling osmotic pressure and acting as a cargo carrier [1,2,3]. In the course of this study, we attempted to reveal its role in the context of pharmacological mobilization of HSPC, a complex process with many interacting partners, which cannot be adequately modeled in vitro. Albumin knockout mice provided the opportunity to address this topic in an in vivo model. Alb−/− mice are healthy; homeostatic hematopoiesis, both immature and mature, is comparable to their WT littermates. Thus, we conclude that albumin has no apparent effect on mature and immature hematopoiesis under physiological conditions. From these observations, specifically normal neutrophil counts, we conclude that endogenous G-CSF, which almost exclusively drives neutrophil production and maturation in the BM, clearly acts independently from serum albumin levels, likely because it is generated within BM (i.e., directly at its site of function). However, neither the role of albumin deficiency during hematopoietic aging, nor on the function of HSC niche in transplantation models, nor radiation protection, were addressed in our study. Thus, possible long-term effects of albumin deficiency on stress hematopoiesis cannot be excluded. In contrast, we show a high impact of albumin in settings of transport of G-CSF, or similar substances, when albumin works as a drug carrier. The two compounds used for clinical stem cell mobilization seem to be strongly influenced by serum albumin concentrations, albeit in opposite directions. AMD3100 mobilizes HSPC by directly targeting CXCR4 [35]. AMD3100 was previously shown to bind to plasma proteins in healthy individuals [31]; up to 60% of circulating AMD3100 is captured. Apparently, the interaction with albumin stabilizes AMD3100, since we could show that in the absence of albumin, 30% of its mobilizing activity is lost. Human serum albumin substitution to physiological levels rescued AMD3100-induced mobilization in Alb−/− mice. rhG-CSF-induced HSPC mobilization, on the other hand, showed a very unique picture in Alb−/− mice. The five-day course of rhG-CSF administration led to a 2-fold higher mobilization efficiency in Alb−/− mice compared to WT littermates. The much elevated rhG-CSF levels in the BM of Alb−/− mice, and the induced proliferation, presumably account for this phenomenon. rhG-CSF not only accumulated more efficiently in the BM of Alb−/− mice after i.p. administration, but its clearance was also delayed in albumin deficient hosts (8-fold more rhG-CSF 4 h after administration in Alb−/− BM). Interestingly, rhG-CSF plasma levels were independent of albumin levels. Thus, in Alb−/− mice, BM resident HPSCs are specifically exposed to higher rhG-CSF levels and the exposure is significantly prolonged compared to WT mice. This, in aggregate, presumably caused enhanced proliferation and higher mobilization efficiency in albumin-deficient mice. This effect is strictly albumin-dependent, as substitution of Alb−/− mice with human serum albumin reduced BM HSPC proliferation, and the number of circulating HSPC after rhG-CSF treatment to normal levels. Regarding the fact that rhG-CSF is used clinically for the mobilization of HSPC in healthy volunteers and in hematologic patients, who typically have largely physiological amounts of serum albumin, we assume fast clearance of rhG-CSF from their BM. Standard regimen is 10 µg/kg/day as a single or split dose (q12h) [14]. Here, we provide evidence supporting the twice-daily injection regimen: Plasma levels of rhG-CSF were already assessed in various studies after a single or multiple injections [36,37,38,39]. However, our data demonstrate that plasma rhG-CSF concentration poorly correlates with rhG-CSF levels in BM (i.e., pharmacologically effective rhG-CSF levels) and thus with mobilization efficacy. We show that rhG-CSF was already barely detectable in the BM of WT mice 4 h after rhG-CSF administration, even though plasma levels remained constant over that time. Seemingly, the prolonged exposure of HSPC to rhG-CSF in the BM of the Alb−/− mice is causal for the observed increase of circulating HSPC, while plasma rhG-CSF concentrations are independent of albumin. Thus, we conclude that BM rhG-CSF concentration more reliably predicts HSPC mobilization efficiency than rhG-CSF plasma levels. Moreover, the prompt drop of rhG-CSF concentration in the BM of WT mice suggests that split-dose rhG-CSF regiment leads to prolonged, essentially continuous rhG-CSF exposure of BM resident HSPC, and thus could explain the observed higher mobilization efficiencies [40]. Efforts have been made to prolong the half-life of rhG-CSF by PEGylation [41]. Studies have shown that single injections of PEG-rhG-CSF were at least as potent as multiple injections of rhG-CSF in treating neutropenia [42]. Fusion of rhG-CSF to an Fc receptor [43] or serum albumin [44,45] was effective in increasing half-life of rhG-CSF and thus mobilization efficiency. In all of these studies, plasma levels of rhG-CSF were considered to be a marker for effectiveness of the modification. Based on our data, we conclude that comparison of the accumulation of those variants in the BM after injection over time would likely lead to a better understanding of rhG-CSF mobilization outcomes.

In the course of this study, we have addressed the impact of serum albumin on homeostatic hematopoiesis and pharmacological mobilization of HSPC. We have shown that homeostatic mature and immature hematopoiesis is independent from albumin. However, when it comes to drug delivery and drug efficacy of clinically relevant mobilizing agents, albumin has marked impact on hematopoietic outcomes, as similarly already shown for many other pharmaceuticals [46,47,48]. Here, we specifically addressed the interactions between albumin and the mobilization drugs AMD3100 and rhG-CSF, showing the dependence of both substances on albumin. In the presence of albumin, rhG-CSF was rapidly cleared out of the BM. Thus, multiple injections per day of rhG-CSF in clinics can be considered reasonable.

## Supplementary Materials

The following are available online at https://www.mdpi.com/2073-4409/9/1/4/s1, Table S1: FACS antibodies used in the study; Figure S1: Immunological 5-lineage differentials reveal no difference between WT and Alb−/− mice; Figure S2: No effect of albumin on cellularity and cell cycle in spleen of rhG-CSF treated mice; Figure S3: Normalization of rhG-CSF induced cell cycle activity of lin- cells in spleens of Alb−/− mice after substitution with hAlb.
