# Supplementary material for: Albumin Modifies Responses to Hematopoietic Stem Cell Mobilizing Agents in Mice
Source: Cells. 2019 Dec 18;9(1):4. doi: 10.3390/cells9010004 (PMC7017167; doi:10.3390/cells9010004)
Supplement: Supplementary file 1 [file cells-09-00004-s001.pdf]

Murine antigen (reactivity)	*Clone*	*Fluorochrome*	*Manufacturer*
7-AAD			BioLegend
CD117 (c-kit)	2B8	APC-Cy.7	BioLegend
CD117 (c-kit)	ACK2	PE-Cy.7	BioLegend
CD11b	M1I70	PE	BioLegend
CD3	17A2	eFluor450	BioLegend
CD45	30-F11	PacificBlue	BioLegend
CD45	30-F11	eFluor450	Invitrogen
CD45R (B220)	RA3-6B2	PE-Cy.7	BioLegend
Gr-1	RB6-8C5	APC	BioLegend
Ki67	SolA15	eFluor450	Invitrogen
Lineage cocktail		PacificBlue	BioLegend
Lineage cocktail		APC	BD Bioscience
Sca-1	D7	BV510	BioLegend
Sca-1	D7	PE	BioLegend
Ter-119	Ter-119	FITC	BioLegend

Table S1. FACS antibodies used in the study

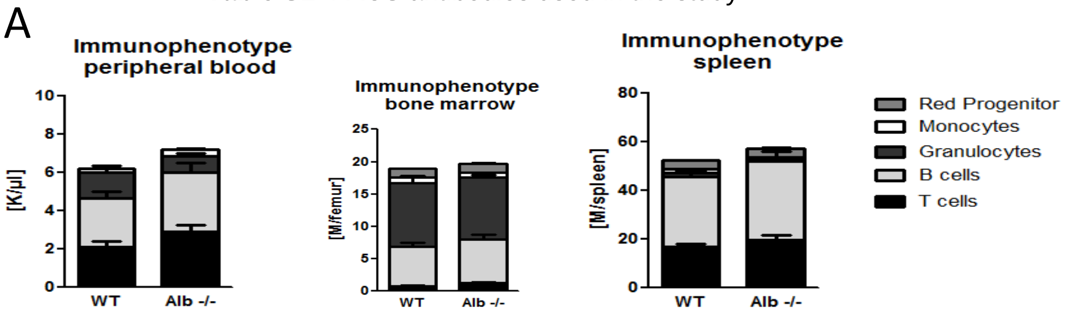

	**WT**	**Alb-/-**
**RBC [M/µL]**	**8.23 ± 0.53**	**9.72 ± 0.50**
**HCT [%]**	**32.06 ± 2.43**	**36.08 ± 1.91**
**PLT [k/µL]**	**578 ± 58**	**716 ± 67**

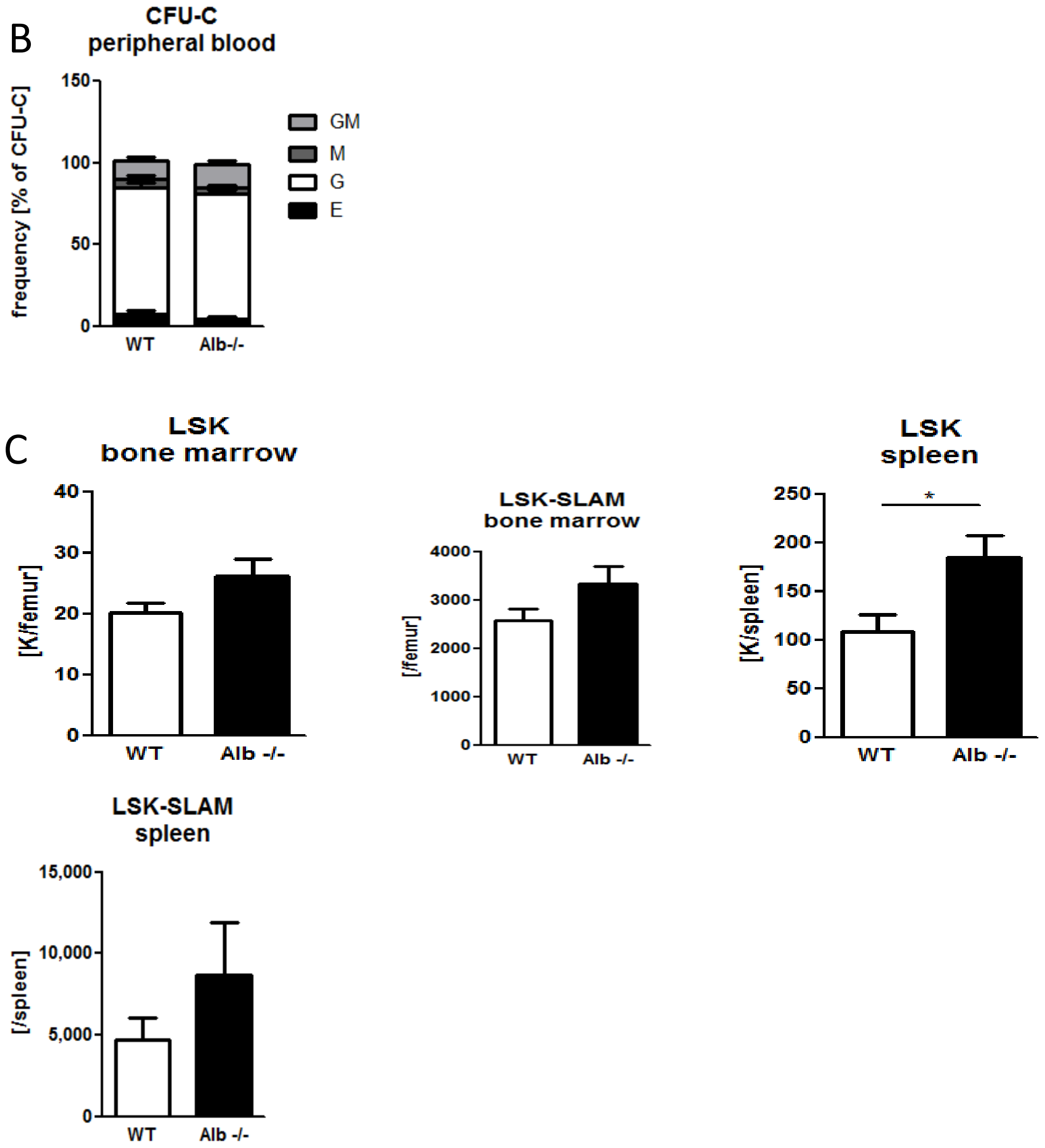

Figure S1: Immunological 5-lineage differentials reveal no difference between WT and Alb-/- mice: Mature WBC and differentials in peripheral blood, BM and spleen were indistinguishable between WT and Alb-/- mice, while Alb-/- mice showed slight polycythemia and thrombocytosis, likely to reduced intravascular osmotic pressure (A) (RBC – red blood cells, HCT – hematocrit, PLT – platelets). CFU-C differential, CFU-C (E-BFU-erythrocyte, G-granulocyte, M-monocyte, GM-granulocyte/monocyte) (B). Immature cells, LSK and LSK-SLAM, were similar in BM but increased in spleens of Alb-/- mice compared to WT mice (C) (n.s. for LSK-SLAM cells). n≥10 mice per group.

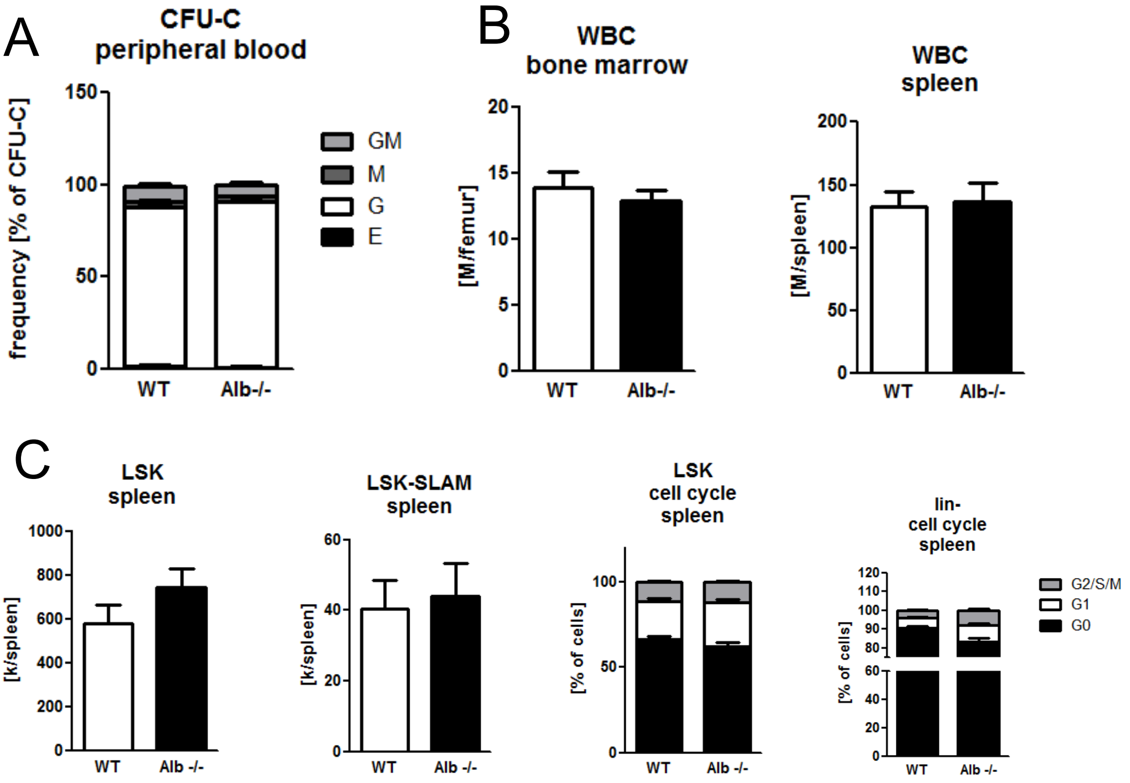

Figure S2: No effect of albumin on cellularity and cell cycle in spleen of rhG-CSF treated mice: No difference in the mobilized CFU-C frequencies between the genotypes; CFU-C (E-BFU-erythrocyte, G-granulocyte, M-monocyte, GM- granulocyte/monocyte) (A) Total WBC counts in BM and spleen (B), total LSK and LSK-SLAM counts (C) as well as LSK and lin- cell cycle were analyzed in rhG-CSF-treated Alb-/- and WT mice (D). Data from 2 independent experiments with n≥3 mice per group.

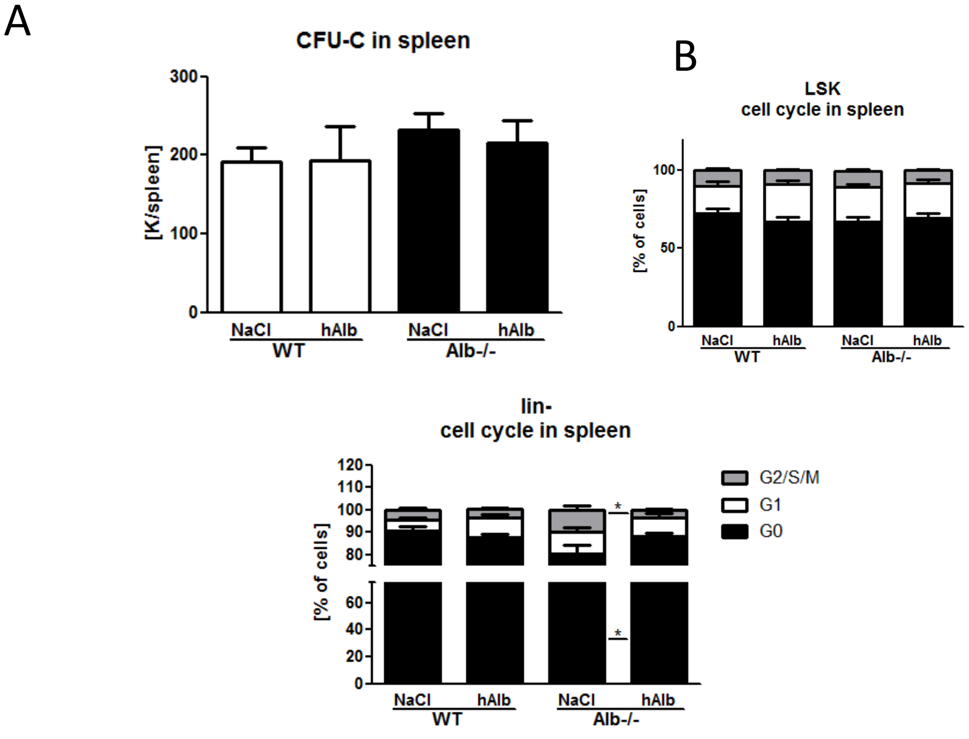

Figure 3. Normalization of rhG-CSF induced cell cycle activity of lin- cells in spleens of Alb-/- mice after substitution with hAlb: CFU-C contents of spleens was indistinguishable in WT or Alb-/- mice treated for 5 days with G-CSF in NaCl or HSA (A); similarly cell cycle activity of LSK cells was

unaffected (B, left), whereas lin- cell cycle activity, enhanced in Alb-/- mice treated with rhG-CSF + NaCl, was normalized when rhG-CSF was supplemented with hAlb (B, right) Data from 3 individual experiments with n≥6 mice per group.
